# Histo-Blood Group Antigens in Children with Symptomatic Rotavirus Infection

**DOI:** 10.3390/v11040339

**Published:** 2019-04-10

**Authors:** Raúl Pérez-Ortín, Susana Vila-Vicent, Noelia Carmona-Vicente, Cristina Santiso-Bellón, Jesús Rodríguez-Díaz, Javier Buesa

**Affiliations:** Department of Microbiology, School of Medicine, University of Valencia and Clinical Microbiology Service, Hospital Clínico Universitario de Valencia, Instituto de Investigación INCLIVA, 46010 Valencia, Spain; raul.perez@clinicalapau.es (R.P.-O.); susana.vila@uv.es (S.V.-V.); noelia.carmona@uv.es (N.C.-V.); cristina.santiso@hotmail.com (C.S.-B.); jesus.rodriguez@uv.es (J.R.-D.)

**Keywords:** rotavirus, histo-blood group antigens (HBGAs), secretor, Lewis, ABO group antigens, susceptibility, gastroenteritis

## Abstract

Group A rotaviruses are a major cause of acute gastroenteritis in children. The diversity and unequal geographical prevalence of rotavirus genotypes have been linked to histo-blood group antigens (HBGAs) in different human populations. In order to evaluate the role of HBGAs in rotavirus infections in our population, secretor status (FUT2+), ABO blood group, and Lewis antigens were determined in children attended for rotavirus gastroenteritis in Valencia, Spain. During three consecutive years (2013–2015), stool and saliva samples were collected from 133 children with rotavirus infection. Infecting viral genotypes and HBGAs were determined in patients and compared to a control group and data from blood donors. Rotavirus G9P[8] was the most prevalent strain (49.6%), followed by G1P[8] (20.3%) and G12P[8] (14.3%). Rotavirus infected predominantly secretor (99%) and Lewis b positive (91.7%) children. Children with blood group A and AB were significantly more prone to rotavirus gastroenteritis than those with blood group O. Our results confirm that a HBGA genetic background is linked to rotavirus P[8] susceptibility. Rotavirus P[8] symptomatic infection is manifestly more frequent in secretor-positive (FUT2+) than in non-secretor individuals, although no differences between rotavirus G genotypes were found.

## 1. Introduction

Group A rotaviruses are the main cause of acute gastroenteritis in infants and young children worldwide, with similar prevalence in developed and developing countries. However, the most severe cases and higher mortality rates occur in developing countries [1]. The World Health Organization (WHO) recommends the use of rotavirus vaccines in all national immunization programs, particularly in South and Southeastern Asia and sub-Saharan Africa [2]. Two oral, live-attenuated rotavirus vaccines (Rotarix and RotaTeq) are available internationally, both considered safe and effective in preventing gastrointestinal disease. However, the efficiency of rotavirus vaccines has been reported to be lower in African children [3,4]. Malnutrition, concomitant infections, simultaneous administration with oral poliovirus vaccine, rotavirus strain diversity, and genetic host factors have been proposed to explain these differences. In addition, the intestinal microbiome may contribute to alter the immune response to rotavirus vaccines [5,6]. It was reported that human rotaviruses recognize the different host histo-blood group antigens (HBGAs) of individuals in a type-specific manner [7,8,9]. HBGAs are complex carbohydrates located on the surface of red blood cells and mucosal epithelia and in body fluids such as saliva, intestinal secretions, milk, and blood as soluble oligosaccharides [10,11,12]. These antigens are genetically determined and depend on an individual’s ABO, secretor, and Lewis status. Recent epidemiological studies indicate that histo-blood group antigens (HBGAs) act as susceptibility factors for the globally dominant P[4], P[6], and P[8] genotypes of human strains of rotavirus A, that recognize fucosylated HBGAs through their spike protein VP8* [13,14,15].

The gene responsible for the secretor phenotype, *FUT2*, encodes an α(1,2)fucosyltransferase that produces the carbohydrate H found on the surface of epithelial cells and in mucosal secretions [16]. The Lewis gene (*FUT3*) codes for an α(1,3/4)fucosyltransferase that transfers fucose to the subterminal βGlcNac unit of precursor chains [17]. Rotavirus P[6] mainly infects Lewis-negative children, a phenotype more common in African populations, providing a plausible explanation for the relatively high frequency of the P[6] genotype in Africa and in some Latin American countries [8,18,19,20]. It was hypothesized that resistance to P[8] strains in Lewis-negative children could be an important contributing factor to the low rotavirus vaccine efficacy in sub-Saharan Africa [8]. However, secretor genotyping in other studies showed that P[8] rotaviruses infect both secretor and non-secretor individuals and that infection correlated with the presence of Lewis antigen [21].

The aim of this study was to evaluate the HBGA profile (ABO, secretor, and Lewis status) in rotavirus-infected children in Valencia, Spain and to investigate potential associations between rotavirus P/G genotypes and HBGA patterns in patients. 

## 2. Materials and Methods

### 2.1. Study Population and Specimens

Stool and saliva samples were collected from 133 children under 5 years of age with rotavirus infection between January 2013 and December 2015. Children were attended at the pediatric clinics and emergency room of the Hospital Clínico Universitario of Valencia (Department of Health No. 5). This study was conducted with the approval of the Ethics Committee of the Hospital (code F-CE-GEva-15; 26 March 2015), and informed written consent was obtained from patients’ parents/tutors before sample collection. Only patients with signed consent were enrolled. To compare the genetic background of the rotavirus-infected children with non-infected counterparts, the distribution of ABO blood groups, H type 1 (*FUT2*), and Lewis antigens were assessed in a control group of 50 healthy children of the same ages and geographic locations. Data from blood donors (*n* = 283,399 individuals) were also obtained from the Transfusion Center of the Autonomous Region of Valencia (Dr. Emma Castro Izaguirre, personal communication).

### 2.2. Rotavirus Detection and Genotyping

Rotaviruses were detected by immunochromatographic assay (Rotavirus–Adenovirus CerTest Biotec, Zaragoza, Spain) and rotavirus G (VP7) and P (VP4) genotypes were determined by a semi-nested multiplex RT-PCR method. For this purpose, a 10%–20% suspension of stool sample was prepared in phosphate buffered saline and subsequent viral RNA extraction was performed using TRIzol (Life Technologies, Carlsbad, CA, USA). Rotavirus G and P genotyping was carried out by RT-PCR following the standardized procedures of the EuroRotaNet network (www.eurorota.net) [22].

### 2.3. Determination of Histo-Blood Group Antigens in Saliva

Lewis (Le^a^ and Le^b^) antigens and ABO group phenotypes were analyzed in saliva samples by enzyme-linked immunosorbent assay (ELISA), essentially as previously described [23]. Polystyrene microtiter plates (Costar, Corning, NY, USA) were coated with previously boiled saliva diluted 1:500 in coating buffer (0.1 M carbonate–bicarbonate buffer, pH 9.6) and incubated for 2 h at 37 °C followed by 4 °C overnight. Plates were washed with phosphate-buffered saline (PBS) containing 0.05% Tween-20 (PBS-T) and blocked with 3% bovine serum albumin (BSA) in PBS. Monoclonal antibodies anti-A and anti-B (Diagast, Loos, France), anti-Le^a^ and anti-Le^b^ (Covance, Dedham, MA, USA), were diluted 1:100 in PBS with 1% BSA and incubated for 1 h at 37 °C. After three washes, horseradish peroxidase goat anti-mouse IgG (Sigma Immunochemicals, St. Louis, MO, USA) diluted 1:2000 in PBS–BSA was added, and incubated for 1 h at 37 °C. After three washes, reactions were developed with *o*-phenylenediamine dihydrochloride (OPD-Fast) (Sigma, St. Louis, MO, USA), stopped with 2M H_2_SO_4_, and recorded at 492 nm. The cutoff value was defined as a threefold increase in absorbance value compared to two negative control samples. 

### 2.4. Genotypic Characterization of the FUT2 Gene (Secretor Status)

Saliva DNA was extracted with a commercial kit (JetFlex Genomic DNA Purification kit, Genomed, Vilnius, Lithuania) and PCR analysis was performed as previously described [16,24]. The secretor genotype (*FUT2*) was characterized by PCR with saliva-extracted DNA and AvaII (Thermo Fisher Scientific, Vilnius, Lithuania) digestion of the amplimers [24,25], to be able to differentiate homozygous and heterozygous alleles for the inactivating mutation G428A in the *FUT2* gene [26]. 

### 2.5. Statistical Analysis

Categorical data were analyzed using the X^2^ test or, when *n* < 5, the Fisher exact test with two-tailed significance was used. Odds ratios (OR) and 95% confidence intervals (CIs) were also calculated. *P* values lower than 0.05 were considered statistically significant. Data were statistically analyzed using R Core Team (2015) v 3.2.2. software. 

## 3. Results

### 3.1. Study Population and Sample Collection

This study was conducted with pediatric patients from the health area served by the Hospital Clínico Universitario of Valencia. The total population attended by this hospital was 345,498, of which 20,091 (5.82%) were children under 5 years of age. Patient ages ranged from 13 days to 5 years, average 22 months. Most children (84.2%) were under 3 years of age, 62 were female (46.6%; 95% CI: 37.9–55.5), and 71 were male (53.4%; 95% CI: 44.5–62.1). A control group composed of 50 healthy children, 24 boys (48%; 95% CI: 33.7–62.6) and 26 girls (52%; 95% CI: 37.4–66.3) with similar demographic characteristics to the patient group was included for comparison.

### 3.2. Rotavirus Genotypes

**Figure 1:**
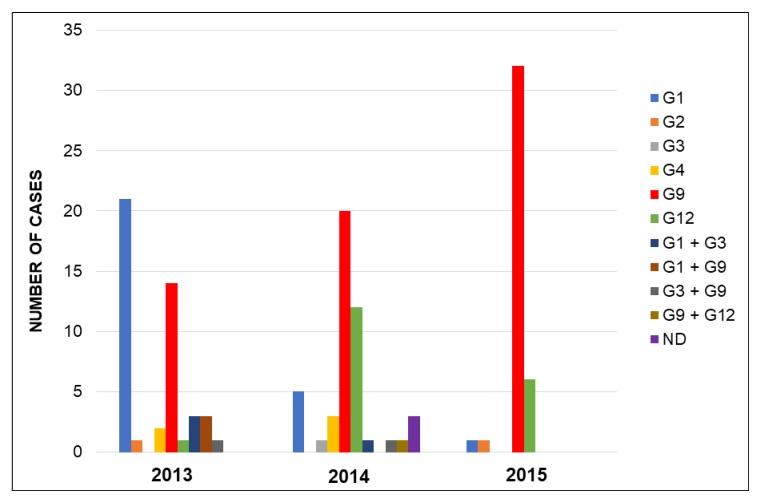
Temporal distribution of rotavirus G genotypes during the three-year study period. Regarding P genotypes, 98% of the strains were P[8] genotype with an overall dominance of G9P[8]. Abbreviations: ND, not determined.

Most children were infected with one genotype (90.2%), 10 (7.5%) children had mixed infections with two genotypes, and in 3 (2.3%) patients the infecting genotype could not be determined. Rotavirus G9P[8] was the most prevalent strain (49.6%), followed by G1P[8] (20.3%) and G12P[8] (14.3%). Other genotypes detected throughout the three-year period were G4P[8] (3.8%), G2P[4] (1.5%), and G3P[8] (0.8%) (Figure 1). Mixed infections caused by G1 + G3P[8] (four cases), G1 + G9P[8] (three cases), G3 + G9P[8] (two cases), and G9 + G12P[8] (one case) were detected. Among the 133 rotavirus strains, 131 were genotype P[8] (97.7%; 95% CI: 93.5–99.5) and only 2 were genotype P[4] (1.5%; 95% CI: 0.2–5.3). No strains of genotype P[6] were detected.

### 3.3. Secretor (FUT2) Status

Rotavirus preferentially infected secretor (98.5%) (95% CI: 94.7–99.8) and Lewis b positive children 92.5% (95% CI: 86.6–96.3) (Table 1). Among the rotavirus-infected secretor individuals, the distribution of homozygous and heterozygous alleles for the *FUT2* gene was 38% and 61%, respectively. In the control group, 70% were secretors and 30% non-secretors (Table 1).

### 3.4. Lewis and ABO Phenotypes

Among the 133 children with rotavirus infection, the phenotypic HBGA distribution was Le^a−b−^ (6.0%), Le^a−b+^ (48.9%), Le^a+b−^ (1.5%), and Le^a+b+^ (43.6%), with 98.5% being secretors (H type 1 positive) and 1.5% non-secretors (Table 1). Distribution of Lewis phenotypes, secretor status, and ABO blood groups in the control group is also shown in Table 1. In our study, children with Lewis b positive phenotype (Le^a−b+^ and Le^a+b+^) (92.5%) were more commonly infected with rotaviruses than those with phenotype Lewis b negative (*p* < 0.05). By contrast, in the control group only 64% individuals were Lewis b positive (Table 1). The percentage of infected children with Lewis a positive phenotype was 45.1% (95% CI: 36.5–54.0), which was lower than the control group (70%; 95% CI: 55.4–82.1) (*p* < 0.05).

The distribution of the ABO blood group phenotypes among rotavirus-infected children was not homogenous, as 37.6% were group O, 48.1% group A, 7.5% group B, and 6.8% group AB (Table 1). This pattern of ABO blood group distribution was similar in the control group (Table 1) and the differences found were not statistically significant (*p* = 0.365). 

### 3.5. Association between HBGAs and Rotavirus Genotypes

The rotavirus genotypes most frequently isolated in our study, G1P[8], G9P[8], and G12P[8], infected more patients with blood groups A and O. However, when we compared the distribution of ABO blood groups in patients to the control group, the difference observed was not statistically significant (*p* = 0.365). When compared with blood donors, group A and AB individuals were at a higher risk of being among gastroenteritis patients than group O individuals (*p* = 0.003) (Table 1). 

G1P[8] and G9P[8] genotypes infected mainly blood group A patients (51.8% and 45.4%, respectively), while the G12P[8] genotype was more frequently isolated in group O patients (47.4%) (Table 2). No significant differences were found in the ABO group antigens among children infected with different G genotypes (*p* = 0.826). All rotavirus genotypes more frequently infected *FUT2* heterozygous individuals, except G12P[8] genotype, which was slightly more prevalent (57.9%) among *FUT2* homozygous individuals (Table 2).

Regarding the patients’ Lewis phenotype no statistically significant differences were found in the distribution of rotavirus G genotypes (*p* = 0.146) (Table 2).

## 4. Discussion

Several studies have related rotavirus susceptibility to human histo-blood group antigens (HBGAs), namely to the secretor status associated with the presence of at least one functional *FUT2* (fucosyltransferase-2) allele, and with Lewis antigens (Le^a^ and Le^b^), which depend on the *FUT3* gene [17,26,27]. These antigens are oligosaccharide compounds made of N-acetyl-glucosamine, galactose, and fucose. The molecular bases for binding the VP8* domain from P[8] VP4 spike protein to its cellular receptor, the secretor H type 1 antigen (Fuc-α1,2-Gal-β1,3-GlcNAc; H1), and to its precursor lacto-*N*-biose (Gal-β1,3-GlcNAc; LNB) have recently been determined [28,29]. Capacity to synthesize secretor H type 1 antigen at the mucosae, determined by the presence of one or two functional copies of the fucosyltransferase *FUT2* gene (secretor status), has been clearly linked to infectivity in other enteric viruses such as the noroviruses [16,26]. However, some controversy existed about the contribution of H1 antigen to rotavirus infection. Epidemiological data has only recently evidenced lower incidence of rotavirus symptomatic infections in non-secretor individuals unable to produce H1 [7], as is also reported in this study.

Several interactions between rotavirus and HBGAs have been described. The recombinant protein VP8* of the genotypes P[4], P[6], and P[8], which belong to the P[II] genogroup, recognize H type 1 antigen. It was previously reported that besides the H type 1 antigen, genotypes P[4] and P[8] could also interact with the Lewis b antigen [7]. However, recent structural data obtained with other members of the P[II] genogroup (genotypes P[4], P[6], P[19]) indicate that the recognition of the VP8* from this genogroup occurs via the type 1 precursor, the lacto-*N*-biose (LNB, Gal β1-3 GlcNac), which interacts within the GlcNac where fucose is added by the FUT3 enzyme [28,29]. These recent discoveries are in contradiction with previous data reported by Huang and collaborators [7]. In addition, the genotypes P[9], P[14], and P[25] of the P[III] genogroup, which infect humans, bind specifically to antigen A [30], and genotype P[11] of the P[IV] genogroup, which infects infants, binds to the type 2 precursor [31]. 

The fact that P[4], P[6], and P[8] rotaviruses recognize the secretor H antigen seems to be related to the higher prevalence of these genotypes worldwide. The secretor antigen is present in 80% of the population of North America and Europe [12]. Genotypes P[4] and P[8] are the most frequently found in human infections worldwide, with a higher prevalence of genotype [P8] [32]. However, the P[6] genotype is more prevalent in Africa, Asia, and in non-African newborns [33,34,35]. Some studies have shown a predominance of secretor Lewis-negative individuals in African, Latin American, and Asian countries, in contrast to North America and Europe, where secretor Lewis-positive individuals predominate [23,26,36]. As in North America and the rest of Europe, most of the Spanish population has a Lewis-positive secretor phenotype (FUT2+), which has been related to infection by genotypes P[4] and P[8], but not by genotype P[6]. This would explain why almost 98% of the rotaviruses detected in this study were P[8] genotypes but none were P[6] genotype. However, in surveys conducted in Burkina Faso, where the majority of the population is Lewis negative, a majority presence of P[6] genotype infections is observed [8]. These Burkina Faso results were interpreted to mean that Lewis b antigen could be a requisite for infection with rotavirus genotypes P[4] and P[8] [8]. Nevertheless, as previously mentioned, this interpretation is not consistent with new, recently reported structural data [28,29]. Something similar happens with non-African neonates; although they are genetically Lewis b positive, expression of these antigens on the surface of erythrocytes can be delayed for the first two months of life, during which time they are Lewis negative [37]. However, it is important to note that these studies were performed in red blood cells, which may not reflect the presence of Lewis antigens on enterocytes and/or in secretions of the intestinal tract. In addition, very little is known about HBGA expression and evolution during development or with concomitant infections in small children. Seven patients in our study got rotavirus infection within the first three months of life, all of them secretor Lewis b positive. The fact that these children express the Lewis b antigen in their mucosae at such a young age has not been observed in other studies [37]. It has been reported that the delay in expression of certain Lewis antigens during the first months of life, despite having an active *FUT3* gene, reinforces the idea that the Lewis antigen might indeed have a preventative effect against rotavirus infection and be part of the age-related restriction factors found in this viral infection.

The G9P[8] genotype proved the most prevalent in our geographical area during the study period with secretor phenotype (FUT2+), as described in other studies carried out in France and Vietnam [36,38]. In our study, the presence of Lewis b antigen in patients (92.5%) was higher than described for the general population of our geographical area, where 69% of individuals are Lewis b positive, estimated from a total of 283,399 individuals (according to unpublished data from the Transfusion Centre of the Autonomous Region of Valencia, Spain). By contrast, 6% of patients were Lewis negative (identical to the 6% described in the control group), but they were nevertheless infected with a P[8] genotype, which contrasts with the results by Nordgren et al. (2014) [8]. This raises the possibility of the secretor H type 1 antigen, rather than the Lewis antigens, being the relevant factor for rotavirus P[8] genotype infection. However, these data must be reinforced by further analyses of P genotypes other than P[8]. It has also been recently reported that the Lewis a phenotype is a restriction factor for RotaTeq and Rotarix vaccine uptake in Nicaraguan children [20]. Moreover, it must be taken into account that aside from HBGA expression, intestinal microbiota composition may play a significant role in susceptibility to rotavirus infections [39]. 

It was suggested that certain rotavirus P genotypes may be prone to infect blood group A individuals [31] and supporting this we found that group A and AB individuals were more commonly found among patients than group O individuals. As expected, we found a lower infection rate among previously vaccinated than unvaccinated patients [40], and observed no differences in the G genotypes of the infecting rotavirus strains and the brand of vaccine administered (Rotarix or RotaTeq).

Interestingly, it has recently been reported that HBGA-binding specificities of human rotaviruses are associated with the disease, but not with in vitro infection [41]. In this regard, further research is needed to clarify how HBGAs determine rotavirus infection and disease in the human host.

## 5. Conclusions

Our results confirm that genetic background leading to different HBGA expression is linked to susceptibility to group A rotavirus symptomatic infection. Rotavirus P[8] infection is manifestly more frequent in secretor (FUT2+) than in non-secretor individuals, although no significant differences between rotavirus G genotypes were found.

## Figures and Tables

**Table 1:** Distribution of histo-blood group antigens (HBGAs) in rotavirus-infected children (*n* = 133), in the control group (*n* = 50), and in blood donors (*n* = 283,399).

	Patients		Controls ^a^		*p* Value ^b^	Odds Ratio ^c^		Donors		*p* Value ^b^
	(*n* = 133) (%)		(*n* = 50) (%)			95% CI		(*n* = 283,399) (%)		
**Blood Group**										
**O**	50	(37.6)	15	(36.6)	0.365	*		146,454	(51.7)	0.003
**A**	64	(48.1)	17	(41.5)		0.89	(0.4–2)	110,273	(38.9)	
**B**	10	(7.5)	7	(17.1)		2.31	(0.7–7.2)	19,054	(6.7)	
**AB**	9	(6.8)	2	(4.9)		0.78	(0.1 - 3.6)	7618	(2.7)	
**Secretor (FUT2)**										
**Secretor**	131	(98.5)	35	(70)	0.000	*		NA		-
**Non-secretor**	2	(1.5)	15	(30)		25	(6.7–100)	NA		
**Lewis (FUT3)**										
**Negative**	8	(6)	3	(6)	1.000	*		31,090	(11)	0.091
**Positive**	125	(94)	47	(94)		0.97	(0.26–4.84)	252,309	(89)	
**Lewis A**										
**Negative**	73	(54.9)	15	(30)	0.005	*		225,219	(79.5)	0.000
**Positive**	60	(45.1)	35	(70)		2.81	(1.42–5.78)	56,180	(20.5)	
**Lewis B**										
**Negative**	10	(7.5)	18	(36)	0.000	*		88,784	(31.3)	0.000
**Positive**	123	(92.5)	32	(64)		0.15	(0.06–0.35)	194,615	(68.7)	
**Lewis A/B**										
**Le a– b–**	8	(6)	3	(6)	0.000	*		31,090	(11)	0.000
**Le a– b+**	65	(48.9)	12	(24)		0.49	(0.1–2.6)	194,129	(68.5)	
**Le a+ b–**	2	(1.5)	15	(30)		16.47	(2.6–169.7)	57,694	(20.3)	
**Le a+ b+**	58	(43.6)	20	(40)		0.9	(0.2–4.6)	486	(0.2)	

Abbreviations: CI, confidence interval; Le, Lewis; NA, not available; ^a^ ABO blood group phenotype was determined in 41 children in the control group; ^b^ X^2^ test with two-tailed significance. When *n* < 5, the Fisher exact test with two-tailed significance was used; ^c^ unadjusted odds ratio; * reference category for the odds ratio estimation.

**Table 2:** Association between ABO blood group, secretor status (secretor, heterozygous/homozygous, non-secretor), and Lewis phenotypes with the infecting G/P rotavirus genotypes.

	G1P[8]		G2P[4]		G3P[8]		G4P[8]		G9P[8]		G12P[8]		*p* Value ^a^
	(*n* = 27) (%)		(*n* = 2) (%)		(*n* = 1) (%)		(*n* = 5) (%)		(*n* = 66) (%)		(*n* = 19) (%)		
**Blood Group**													
**O**	10	(37)	0	(0)	0	(0)	2	(40)	24	(36.4)	9	(47.4)	0.826
**A**	14	(51.8)	2	(100)	1	(100)	3	(60)	30	(45.4)	7	(36.8)	
**B**	2	(7.4)	0	(0)	0	(0)	0	(0)	7	(10.6)	1	(5.3)	
**AB**	1	(3.7)	0	(0)	0	(0)	0	(0)	5	(7.6)	2	(10.5)	
**Secretor**													
**Heterozygous**	16	(59.3)	2	(100)	1	(100)	3	(60)	44	(66.7)	8	(42.1)	0.265
**Homozygous**	10	(37)	0	(0)	0	(0)	2	(40)	22	(33.3)	11	(57.9)	
**Non-secretor**	1	(3.7)	0	(0)	0	(0)	0	(0)	0	(0)	0	(0)	
**Lewis A / B**													
**Le a– b–**	4	(50)	0	(0)	0	(0)	0	(0)	2	(25)	1	(12.5)	0.146
**Le a– b+**	15	(23.1)	1	(1.5)	0	(0)	3	(4.6)	30	(46.1)	11	(16.9)	
**Le a+ b–**	1	(50)	0	(0)	0	(0)	0	(0)	0	(0)	0	(0)	
**Le a+ b+**	7	(12.1)	1	(1.7)	1	(1.7)	2	(3.4)	34	(58.6)	7	(12.1)	

^a^ X^2^ test with two-tailed significance. When *n* < 5, the Fisher exact test with two-tailed significance was used.
